# A Note on the Effects of Yoghurt Acid Whey Marination on the Tenderness and Oxidative Stability of Different Meat Types

**DOI:** 10.3390/foods10112557

**Published:** 2021-10-23

**Authors:** Panagiotis Simitzis, Fotini Zikou, Dionisis Progoulakis, Georgios Theodorou, Ioannis Politis

**Affiliations:** Laboratory of Animal Breeding and Husbandry, Department of Animal Science, Agricultural University of Athens, 75 Iera Odos, 11855 Athens, Greece; fotinizikou96@hotmail.com (F.Z.); dionisispro@gmail.com (D.P.); gtheod@aua.gr (G.T.); i.politis@aua.gr (I.P.)

**Keywords:** yoghurt acid whey, pork meat, lamb meat, chicken meat, rabbit meat, shear force, color parameters, antioxidant activity

## Abstract

The aim of this preliminary study was to examine the effects of yoghurt acid whey (YAW) marination on quality parameters and the oxidative stability of pork, lamb, rabbit and chicken meat. Twenty-four samples per meat type were randomly allocated to one of four groups: CON, without any treatment; YAW1 and YAW2, where samples were marinated for 20 h at 4 °C at a pH of 5 or 4.5, respectively; and YAW3, where samples were treated as in the YAW2 group except hesperidin was also added at the level of 2 g/L. As indicated, meat tenderness was improved as a result of YAW marination, apart from the chicken samples. In general, values of pH, redness and yellowness were decreased after immersion in YAW both in raw and cooked samples. However, lightness was increased in the raw meat samples as a result of YAW marination, though this effect was not observed in the cooked meat samples with the exception of chicken meat. Chroma values were higher in controls compared to YAW-treated groups in raw pork and lamb meat, while no significant differences regarding chroma were found among groups in cooked lamb and rabbit meat. Hue angle values were greater in YAW-treated groups compared to controls in raw samples, whereas no significant differences among groups were indicated in cooked meat. Meat oxidation rates were not affected by treatment with YAW and the hesperidin addition, which improved the oxidative stability of lamb and chicken meat. Thus, YAW marination could be recommended as a novel strategy that improves meat tenderness without negative effects on the other quality characteristics.

## 1. Introduction

Yoghurt production has continually increased around the world, from 24,016 tn in 1994 to 61,821 tn in 2004 to 163,593 tn in 2014, according to FAO [1]. As a result, larger quantities of yoghurt acid whey (YAW) or serum are also produced, which pose a greater pollution risk for the environment compared to previous decades. YAW is derived using a centrifugal separator or a filtration membrane after the fermentation of yoghurt, and can cause problems for dairy companies since it demands a special treatment due to its high organic matter content before being discharged into streams [2].

In contrast to the sweet whey derived from cheese production, YAW has lower levels of protein and higher concentrations of lactic acid and calcium [3,4]. The use of sweet whey in a variety of food systems as a flavor enhancer, egg white substitute and binder has been firmly established during the recent decades [5]. The abundance of lactic acid and calcium in YAW may lead to its regular application in the meat industry as a tenderizing agent. According to the existing literature, the incorporation of acid whey in sausages [6] and dry-cured pork loins [7] did not have a negative effect on their physicochemical characteristics and sensory attributes. At the same time, lactic acid and calcium solutions have been found to be effective in increasing beef meat tenderness [8,9]. Tenderness is one of the primary sensory attributes that consumers associate with meat palatability, so it drastically affects their choice. As a result, the meat industry is continuously striving to find ways of providing more tender meat products; one possible method of accomplishing this goal is acid marination [10]. 

Data regarding the effects of YAW marination on meat characteristics are scarce. The aim of this preliminary experiment was therefore to highlight possible differences in quality traits and the oxidative stability of different types of meat as an effect of yoghurt acid whey marination.

## 2. Materials and Methods

### 2.1. Samples and Treatments

Different meat types were obtained from a local supermarket in Athens, transported to the laboratory on ice and stored at 4 °C until their analysis within the same day. For the quality assessment analyses, the section of longissimus thoracis muscle between the 6th and 13th ribs was used for pork and lamb, the pectoralis major muscle for chicken and the longissimus lumborum muscle for rabbit meat. Meat samples were derived from six animals per meat type with each sample further divided into four sub-samples for the respective experimental groups. Each sub-sample per meat type was randomly allocated to one of the following four groups: control (C), without any treatment or one of the three marination treatments; YAW1, where the samples remained in an acid whey marinade with a pH of 5 for 20 h at 4 °C; YAW2, where the samples remained in an acid whey marinade with a pH of 4.5 for 20 h at 4 °C; and YAW3, where the samples remained in an acid whey marinade with a pH of 4.5 for 20 h at 4 °C with a concomitant addition of hesperidin (TSI Europe NV, Zwijndrecht, Belgium) at the level of 2 g/L. Hesperidin is a health-promoting compound with multifunctional biological properties and an intense antioxidant effect. It is present in citrus fruits, which constitute a great category of Greek flora and contain one or more aromatic hydroxyl groups that actively scavenge free radicals exerting a strong antioxidant activity [11]. 

YAW used in the present study originated from cow milk that was collected from several small dairy farms around Greece. Lactic acid marinade was prepared by adding yoghurt serum powder into distilled water to the desired pH (10 g and 2.25 g YAW per 100 mL for reaching pH values of 4.5 and 5, respectively). Yoghurt serum was mechanically derived after the fermentation of authentic Greek-style strained yoghurt with Streptococcus thermophiles and Lactobacillus bulgaricus in a dry free-flowing powder containing 60% lactose, 12.5% galactose, 5.3% lactic acid and 5.1% protein plus 24.7 g Potassium, 18 g Calcium, 14.4 g Chloride, 6.6 g Sodium, 6 g Phosphorus, 1.7 g Magnesium, 1.13 mg Ferrum and 0.48 mg Copper per kg (Makedonian Protein SA, Kilkis, Greece).

### 2.2. Meat Quality Characteristics 

#### 2.2.1. Tenderness

Raw meat samples (80 ± 2 g) were placed in plastic bags and cooked in a water bath at the following conditions according to meat type: 50 min at 75 °C for pork [12], 35 min at 75 °C for lamb [13], 30 min at 80 °C for chicken [14] and 30 min at 70 °C for rabbit [15] meat. All samples were left under running water for 30 min and then placed in a refrigerator at about 4 °C for 24 h. Five sub-samples with a cross section of 1 cm^2^ were cut parallel to the muscle fibers and the shear force value of the muscle samples was measured using a Warner Bratzler (WB) shear blade fitted to a Zwick Testing Machine Model Z2.5/TN1S (Zwick GmbH & Co., Ulm, Germany). Peak force values in Newton were recorded. Warner–Bratzler shear blade specifications include: crosshead speed set at 200 to 250 mm/min, blade thickness 1.016 mm, v-shaped (60 degree angle) cutting blade, cutting edge beveled to a half-round, corner of the v rounded to a quarter-round of a 2.363 mm diameter circle and 2.032 mm thick spacers providing the gap for the cutting blade to slide through.

#### 2.2.2. pH and Color Parameters

Acidity (pH) values were determined by a meat pH meter (HI 99163 model, Hanna instruments, Nusfalau, Romania) with the electrode inserted into raw and cooked meat samples. Calibration of the pH meter was performed at pH 4.0 and 7.0 (Merck, Darmstadt, Germany) at ambient temperature. Three color measurements per raw or cooked meat sample were implemented at room temperature (~20 °C) using a Miniscan XE (HunterLab, Reston, VA, USA) chromameter set on the L* (lightness), a* (redness) and b* (yellowness) systems (CIE 1976, Commission International de l’ Eclairage). Standardization was performed with a white and a black tile. Chroma and hue angle were also calculated according to the following formulas: chroma = (a^2^ + b^2^)^1/2^ and hue angle = arctan (b*/a*).

#### 2.2.3. Oxidative Stability

Lipid oxidation was evaluated on the basis of the malondialdehyde (MDA) values determined by a selective third-order derivative spectrophotometric method [16]. Briefly, 2 g from each raw or cooked meat sample (2 replications) was homogenized (Edmund Buehler 7400 Tuebingen/H04, Tübingen, Germany) in the presence of 8 mL aqueous trichloroacetic acid (TCA) (50 g/L) and 5 mL butylated hydroxytoluene (BHT) in hexane (8 g/L), and then the mixture was centrifuged for 3 min at 3000× *g*. After the removal of the top hexane layer, a 2.5 mL aliquot from the bottom layer was mixed with 1.5 mL aqueous 2-thiobarbituric acid (TBA) (8 g/L) and was further incubated at 70 °C for 30 min. The mixture was then left under tap water to cool and a third-order derivative (3D) spectrophotometry (Hitachi U3010 Spectrophotometer) in the range of 500–550 nm was applied. Meat MDA levels (ng/g wet tissue) were determined on the basis of the height of the third-order derivative peak at 521.5 nm, by contrasting with the intercept data and slope of the standard calibration curve prepared using the MDA precursor 1,1,3,3-tetraethoxypropane (TEP).

### 2.3. Statistical Analysis

Meat quality characteristics such as pH, color parameters (L*, a*, b*, chroma and hue angle) and malondialdehyde (MDA) (ng/g) values were subjected to an analysis of variance with the treatments of marination (CON, YAW1, YAW2 or YAW3), meat type (raw or cooked) and their interaction as fixed effects. Shear force value (N) was also subjected to an analysis of variance with the treatment of marination (CON, YAW1, YAW2 or YAW3) as a fixed effect. Results are presented as least squares (LS) mean ± standard error of mean (S.E.M.). Mean differences were tested at a 0.05 significance level with Bonferroni adjustment; analyses were performed by Sas/Stat [17].

## 3. Results

As shown in Table 1, meat tenderness was generally improved since shear force values were decreased after YAW marination. The effect was significant in pork, lamb and rabbit meat samples (*p* < 0.01), whereas no significant effect was observed in chicken meat (*p* > 0.05). Significant differences in pH values were observed for cooked pork (*p* < 0.001), cooked lamb (*p* < 0.01), raw rabbit (*p* < 0.001) and raw and cooked chicken (*p* < 0.01) meat. In general, YAW marination caused a reduction in meat pH values.

## 4. Discussion

As shown in the present preliminary study, the high amounts of lactic acid and calcium in YAW decreased the shear force values in pork, lamb and rabbit meat samples. In contrast, Wojciak et al. [18] did not detect any effect of marination in acid whey for 24 h on beef tenderness. However, beef tenderness is generally improved after marination with lactic acid [8,19] or calcium salts [9]. Similar findings were also observed in pork meat treated with lactic acid aqueous solutions [20]. In turkey meat, YAW marination for 12 h decreased the meat shear force values [21]. The mechanism of muscle tissue tenderization by organic acid solutions or calcium salts is still not completely understood. However, the effect of acid marination on meat tenderness is mainly attributed to a loosening of the structure in collagen connective tissue; since the acid-labile cross linkages in the collagen molecule are released, a connective tissue breakdown is caused (especially perimysial tissue degradation) [10,19]. Yet, no effect of YAW marination on chicken breast meat tenderness was observed. This finding is possibly related to the fact that marination appeared to be effective in tenderizing muscles containing a large amount of connective tissue [10].

In general, YAW marination caused a reduction in meat pH values, with significant differences in cooked pork, cooked lamb, raw rabbit and raw and cooked chicken meat. A significant decrease in pH values due to lactic acid marination has also been demonstrated in beef [8] and pork [20] meat. In turkey meat, immersion into YAW decreased pH values in raw and roasted samples [21]. Wojciak et al. [22] reached the same conclusions regarding fermented beef marination in acid whey. Moreover, the addition of acid whey in dry-cured pork loins caused a significant drop in pH values [7].

Meat color parameters were affected by YAW marination. As indicated, the lower pH values of the acid-treated samples may have resulted in the denaturation of sarcoplasmic and myofibrillar proteins, which may alter their water-binding ability. The differences in the amount of water dispersed among the muscle fibers could influence the reflectance ability of the meat [19]. Values for lightness were increased in marinated raw meat samples but this effect was not evident in cooked samples with the exception of chicken meat, where significantly higher L* values were also observed in cooked samples of the YAW3 group compared to the controls. This is very important especially for pre-cooked meat products, since the initial pale color of YAW-treated raw samples is lost due to cooking and the cooked samples have similar lightness. In contrast, values for redness (a*) were decreased after marination in raw meat samples; this reduction was also observed in cooked pork and chicken but not in lamb and rabbit meat samples. Increases in L* values and decreases in a* values were also observed in beef cuts marinated into a lactic acid solution [19,23]. In contrast, an increase in a* and b* values was observed after immersion in fermented beef eye round in acid whey [22]. On the other hand, no effect on color parameters was observed in beef marinated into YAW [18]. Yellowness (b*) in lamb and rabbit samples was not affected by the treatment with yoghurt acid whey. However, a significant reduction in b* values of raw and cooked pork and chicken meat samples was shown after YAW marination. As previously indicated, lactic acid marination of beef did not affect b values [19]. Immersion of turkey meat into YAW increased L* and b* values in raw and roasted samples while a* values were not affected [21]. Moreover, the addition of acid whey in non-nitrite cooked pork sausages decreased yellowness [6]. Immersion of pork meat in a lactic acid solution did not have a significant effect on its color parameters (L*, a*, b*) [20]. 

Finally, YAW marination did not accelerate the oxidation rates of meat samples since the MDA values among CON, YAW1 and YAW2 treatments were similar. This finding was not in agreement with that of Wojciak et al. [18], who found increased values for 2-thiobarbituric acid reactive substances (TBARS) in beef marinated into acid whey after 1 and 28 days but decreased MDA levels after 14 days of storage. At the same time, fermented beef marination in acid whey improved oxidative stability during storage [22]. Moreover, as indicated in the present study, inclusion of hesperidin into a marinade caused a decrease in the MDA values in raw and cooked lamb and chicken meat samples. Hesperidin is a bioactive compound that is contained in citrus fiber and can actively scavenge free radicals [24]. Citrus by-products (lemon albedo or citrus fiber) have already been added to meat products (cooked and dry-cured sausages, cooked turkey meat, mortadella, etc.) with positive effects on their lipid oxidation indices [25,26,27].

## 5. Conclusions

The results of the present preliminary study clearly demonstrated that the marination of different meat types with yoghurt acid whey increased tenderness without negative effects on the other quality traits. The exploitation of this yoghurt processing by-product within the meat industry can lead to a reduction in hazards that are related to its disposal, while an improvement of meat tenderness, without further side effects on the other quality traits, is observed.

## Figures and Tables

**Table 1:** Effects of YAW marination on quality characteristics and oxidative stability of different raw and cooked meat types (LS means ± s.e.m.).

	Raw				Cooked					*p*-Value ^7^		
	CON ^1^	YAW1	YAW2	YAW3	CON	YAW1	YAW2	YAW3	SEM	T	MT	T × MT
Pork (*longissimus thoracis* muscle between the 6th and 13th rib)												
SF ^2^	-	-	-	-	22.57 ^d^	16.12 ^ef^	17.19 ^df^	12.81 ^df^	1.52	<0.01	-	-
pH	6.23	6.12	5.95	6.17	6.46 ^e^	6.51 ^e^	5.98 ^f^	6.11 ^f^	0.06	<0.001	<0.001	<0.01
L* ^3^	39.57 ^a^	55.70 ^c^	50.80 ^b^	49.39 ^b^	54.73	54.13	53.29	53.76	1.15	<0.001	<0.001	<0.001
a*	12.15 ^a^	5.87 ^c^	7.15 ^bc^	7.41 ^b^	4.36 ^df^	4.60 ^f^	3.01 ^d^	2.84 ^e^	0.36	<0.001	<0.001	<0.001
b*	12.57 ^a^	10.28 ^b^	11.76 ^ab^	11.33 ^ab^	16.41 ^d^	15.41 ^df^	13.40 ^ef^	12.17 ^e^	0.60	<0.01	<0.001	<0.001
Chr ^4^	17.49 ^a^	11.85 ^b^	13.83 ^b^	13.55 ^b^	16.01 ^d^	17.05 ^d^	13.75 ^e^	12.50 ^e^	0.61	<0.001	0.140	<0.001
H ^5^	45.94 ^a^	60.15 ^b^	58.81 ^b^	56.65 ^b^	74.20	74.25	77.55	77.15	1.37	<0.001	<0.001	<0.001
MDA ^6^	76.88	72.49	79.47	74.28	137.3	105.9	104.8	93.64	8.20	<0.01	<0.01	0.123
Lamb (*longissimus thoracis* muscle between the 6th and 13th rib)												
SF	-	-	-	-	19.48 ^d^	14.62 ^f^	13.73 ^f^	12.42 ^f^	0.85	<0.001	-	-
pH	5.65	5.64	5.63	5.66	6.05 ^d^	6.02 ^d^	5.93 ^e^	5.89 ^e^	0.02	<0.01	<0.001	<0.01
L*	45.09 ^a^	59.07 ^b^	57.05 ^b^	58.12 ^b^	57.83	59.40	59.43	57.32	0.94	<0.001	<0.001	<0.001
a*	10.54 ^a^	5.43 ^b^	4.94 ^b^	4.71 ^b^	3.00	2.96	2.43	2.55	0.26	<0.001	<0.001	<0.001
b*	13.40	12.93	12.25	12.84	12.32	12.38	11.33	12.99	0.61	0.245	0.175	0.755
Chr	17.05 ^a^	14.02 ^b^	13.22 ^b^	13.68 ^b^	12.69	12.73	11.59	13.24	0.64	<0.01	<0.001	<0.05
H	51.83 ^a^	67.13 ^b^	68.12 ^b^	69.91 ^b^	76.32	76.59	78.00	78.97	0.73	<0.001	<0.001	<0.001
MDA	153.1 ^a^	134.4 ^a^	137.3 ^a^	89.03 ^b^	8707 ^d^	8920 ^d^	9291 ^d^	6324 ^e^	364.5	<0.001	<0.001	<0.001
Rabbit (*longissimus lumborum* muscle)												
SF	-	-	-	-	17.80 ^d^	13.38 ^f^	13.32 ^f^	11.96 ^f^	0.93	<0.01	-	-
pH	5.75 ^a^	5.66 ^b^	5.56 ^c^	5.57 ^c^	6.18	6.13	6.12	6.12	0.02	<0.01	<0.001	<0.001
L*	56.42 ^a^	64.19 ^b^	63.48 ^b^	65.24 ^b^	66.77	67.09	67.20	70.69	1.32	<0.001	<0.001	<0.05
a*	4.96 ^a^	2.81 ^b^	3.22 ^b^	2.88 ^b^	1.48 ^d^	0.88 ^e^	0.99 ^de^	1.50 ^d^	0.28	<0.001	<0.001	<0.01
b*	11.61	10.14	11.11	11.62	12.81	11.75	11.34	13.62	0.61	<0.05	<0.01	0.519
Chr	12.63	10.54	11.58	11.99	12.90	11.78	11.38	13.72	0.62	<0.05	0.091	0.395
H	66.72 ^a^	74.41 ^b^	73.95 ^b^	76.41 ^b^	83.51	85.74	85.01	83.54	1.26	<0.001	<0.001	<0.01
MDA	103.36	164.78	186.46	95.77	378.16	467.47	469.89	240.67	59.47	<0.05	<0.01	0.540
Chicken (*pectoralis major* muscle)												
SF	-	-	-	-	13.47	13.93	13.59	14.78	1.03	0.114	-	-
pH	6.09 ^a^	6.01 ^a^	5.87 ^b^	5.82 ^b^	6.20 ^d^	6.19 ^d^	6.15 ^d^	5.97 ^e^	0.03	<0.001	<0.001	0.061
L*	56.55 ^a^	66.59 ^c^	62.82 ^b^	62.14 ^b^	75.85 ^d^	77.69 ^de^	77.14 ^de^	78.64 ^d^	0.53	<0.001	<0.001	<0.001
a*	4.54 ^a^	1.54 ^c^	3.30 ^b^	2.51 ^b^	1.96 ^d^	1.22 ^e^	1.50 ^de^	1.21 ^e^	0.23	<0.001	<0.001	<0.001
b*	13.38 ^a^	11.24 ^b^	13.46 ^a^	13.33 ^a^	18.10 ^d^	16.16 ^e^	17.42 ^d^	17.88 ^d^	0.35	<0.001	<0.001	<0.01
Chr	14.14 ^a^	11.35 ^b^	13.90 ^a^	13.58 ^a^	18.16 ^d^	16.21 ^e^	17.53 ^d^	19.93 ^e^	0.33	<0.001	<0.001	<0.01
H	71.28 ^a^	82.21 ^c^	76.04 ^ab^	79.26 ^bc^	85.27	85.69	83.59	86.48	1.03	<0.001	<0.001	<0.001
MDA	80.55 ^a^	74.83 ^ab^	75.98 ^ab^	59.06 ^b^	88.36 ^e^	70.71 ^de^	67.77 ^d^	54.29 ^d^	5.12	<0.001	0.526	0.437

^1^ CON: control, without supplementation; YAW1: marination into acid whey with a pH of 5 for 20 h at 4 °C; YAW2: marination into acid whey with a pH of 4.5 for 20 h at 4 °C; YAW3: marination into acid whey with a pH of 4.5 for 20 h at 4 °C with a concomitant addition of hesperidin (2 g/L). ^2^ Shear force value in Newton. ^3^ L*, a*, b*: lightness, redness and yellowness, respectively. ^4^ Chroma = (a^2^ + b^2^)^1/2^. ^5^ Hue angle = arctan (b*/a*). ^6^ Malondialdehyde levels in ng/g (higher levels of MDA indicate increased rates of oxidation). ^7^ T: effect of treatment (CON, YAW1, YAW2 or YAW3); MT: effect of meat type (raw or cooked); T × MT: interaction of treatment by meat type. ^abc^ LS Means within a row where raw meat is not sharing a common superscript letter are significantly different (*p* < 0.05). ^def^ LS Means within a row where cooked meat is not sharing a common superscript letter are significantly different (*p* < 0.05).

## Data Availability

The data presented in this study are available on request from the corresponding author.

## References

[1] FAOSTAT. http://www.fao.org/faostat/en/#data/QP.

[2] Alonso S., Herrero M., Rendueles M., Diaz M. (2010). Residual yoghurt whey for lactic acid production. Biomass Bioenerg..

[3] Menchik P., Zuber T., Zuber A., Moraru C.I. (2019). Composition of coproduct streams from dairy processing: Acid whey and milk permeate. J. Dairy Sci..

[4] Rocha-Mendoza D., Kosmerl E., Krentz A., Zhang L., Badiger S., Miyagusuku-Cruzado G., Mayta-Apaza A., Giusti M., Jimenez-Flores R., García-Cano I. (2020). Invited review: Acid whey trends and health benefits. J. Dairy Sci..

[5] Walzem R.L., Dillard C.J., German J.B. (2002). Whey Components: Millennia of Evolution Create Funcionalities for Mammalian Nutrition: What we know and what we may be overlooking. Crit. Rev. Food Sci..

[6] Wójciak K.M., Karwowska M., Dolatowski Z.J. (2014). Use of acid whey and mustard seed to replace nitrites during cooked sausage production. Meat Sci..

[7] Stadnik J., Stasiak D.M. (2016). Effect of acid whey on physicochemical characteristics of dry-cured organic pork loins without nitrite. Int. J. Food Sci. Technol..

[8] Aktaş N., Aksu M.I., Kaya M. (2003). The effect of organic acid marination on tenderness, cooking loss and bound water content of beef. J. Muscle Foods.

[9] Ostoja H., Cierach M. (2003). Effect of calcium ions on the solubility of muscular collagen and tenderness of beef meat. Nahrung.

[10] Wenham L.M., Locker R.H. (1976). The Effect of Marinading on Beef. J. Sci. Food Agric..

[11] Erlund I. (2004). Review of the flavonoids quercetin, hesperetin, and naringenin. Dietary sources, bioactivities, bioavailability, and epidemiology. Nutr. Res..

[12] Van Oeckel M.J., Warnants N., Boucqué C.V. (1999). Pork tenderness estimation by taste panel, Warner–Bratzler shear force and on-line methods. Meat Sci..

[13] Hopkins D.L., Toohey E.S., Warner R.D., Kerr M.J., Van de Ven R. (2010). Measuring the shear force of lamb meat cooked from frozen samples: Comparison of two laboratories. Anim. Prod. Sci..

[14] Cason J.A., Lyon C.E., Papa C.M. (1997). Effect of muscle opposition during rigor on development of broiler breast meat tenderness. Poult. Sci..

[15] Honikel K.O. (1998). Reference methods for the assessment of physical characteristics of meat. Meat Sci..

[16] Botsoglou N.A., Fletouris D.J., Papageorgiou G.E., Vassilopoulos V.N., Mantis A.J., Trakatellis A.G. (1994). A rapid, sensitive and specific thiobarbituric acid method for measuring lipid peroxidation in animal tissues, food and feedstuff samples. J. Agric. Food Chem..

[17] Sas/Stat (2011). Statistical Analysis Systems, Version 9.3.

[18] Wójciak K.M., Kęska P., Okoń A., Solska E., Libera J., Dolatowski Z.J. (2018). The influence of acid whey on the antioxidant peptides generated to reduce oxidation and improve colour stability in uncured roast beef. J. Sci. Food Agric..

[19] Aktaş N., Kaya M. (2001). The influence of marinating with weak organic acids and salts on the intramuscular connective tissue and sensory properties of beef. Eur. Food Res. Technol..

[20] Grajales-Lagunes A., Rivera-Bautista C., Ruiz-Cabrera M., Gonzalez-Garcia R., Ramirez-Telles J., Abud-Archila M. (2012). Effect of lactic acid on the meat quality properties and the taste of pork Serratus ventralis muscle. Agric. Food Sci..

[21] Augustyńska-Prejsnar A., Hanus P., Sokołowicz Z., Kačániová M. (2021). Assessment of technological characteristics and microbiological quality of marinated turkey meat with the use of dairy products and lemon juice. Anim. Biosci..

[22] Wójciak K.M., Krajmas P., Solska E., Dolatowski Z.J. (2015). Application of acid whey and set milk to marinate beef with reference to quality parameters and product safety. Acta Sci. Pol. Technol. Aliment..

[23] Jimenez-Villarreal J.R., Pohlman F.W., Johnson Z.B., Brown Jr A.H. (2003). Lipid, instrumental color and sensory characteristics of ground beef produced using trisodium phosphate, cetylpypiridinium chloride, chlorine dioxide or lactic acid as multiple antimicrobial interventions. Meat Sci..

[24] Marin F.R., Soler-Rivas C., Benavente-Garcia O., Castillo J., Perez-Alvarez J.A. (2007). By-products from different citrus processes as a source of customized functional fibres. Food Chem..

[25] Fernandez-Lopez J., Fernandez-Gines J.M., Aleson-Carbonell S.E., Sayas-Barbera E., Perez-Alvarez J.A. (2004). Application of functional citrus by-products to meat products. Trends Food Sci. Technol..

[26] Contini C., Alvarez R., O’Sullivan M., Dowling D.P., Gargan S.O., Monahan F.J. (2014). Effect of an active packaging with citrus extract on lipid oxidation and sensory quality of cooked turkey meat. Meat Sci..

[27] Viuda-Martos M., Ruiz-Navajas Y., Fernandez-Lopez J., Perez-Alvarez J.A. (2010). Effect of added citrus fibre and spice essential oils on quality characteristics and shelf-life of mortadella. Meat Sci..

